# Salud mental materna: factor de riesgo del bienestar socioemocional en niños mexicanos

**DOI:** 10.26633/RPSP.2017.1

**Published:** 2017-02-02

**Authors:** César Villaseñor, Jaqueline Calderón Hernández, Efraín Gaytán, Silvia Romero, Fernando Díaz-Barriga

**Affiliations:** 1 Universidad Autónoma de San Luis Potosí Programa Multidisciplinario en Ciencias Ambientales San Luis PotosíSan Luis Potosí México Programa Multidisciplinario en Ciencias Ambientales, Universidad Autónoma de San Luis Potosí, San Luis Potosí, San Luis Potosí, México.; 2 Facultad de Medicina-CIACYT Universidad Autónoma de San Luis Potosí San Luis PotosíSan Luis Potosí México Universidad Autónoma de San Luis Potosí, Facultad de Medicina-CIACYT, San Luis Potosí, San Luis Potosí, México.; 3 Facultad de Psicología Universidad Autónoma de San Luis Potosí San Luis PotosíSan Luis Potosí México Universidad Autónoma de San Luis Potosí, Facultad de Psicología, San Luis Potosí, San Luis Potosí, México.

**Keywords:** Salud mental, mujeres, depresión, ansiedad, desarrollo infantil, México, Mental health, women, depression, anxiety, child development

## Abstract

**Objetivos.:**

Estimar la prevalencia de síntomas depresivos y de ansiedad (SDyA) en madres de tres zonas poblacionales (ZP) del estado de San Luis Potosí, México, y analizar la asociación entre los SDyA y sus efectos en la salud emocional de sus hijos.

**Métodos.:**

Se evaluaron 173 parejas (madre-hijo) de tres ZP: Zona urbana (ZU), rural (ZR) y rural indígena (ZRI). Se estudiaron los SDyA de las mujeres, y las dificultades conductuales (DC) de los niños.

**Resultados.:**

Se encontró una alta prevalencia de SDyA en las tres ZP. La mayor proporción de síntomas depresivos la presentaron la ZU y la ZR (38,7 y 38,6%). La prevalencia más alta de síntomas de ansiedad se observó en la ZR (31,8%). La asociación entre los SDyA y la depresión materna más fuerte se estimó en la ZR (razón de momios o RM = 11,0; IC95%: 1,3-95,5). En la ZRI se estimó la mayor prevalencia de DC en los niños (61%). Se encontró una asociación entre las DC y la ansiedad materna (RM = 2,2; IC95%: 1,1-4,3) y la depresión materna (RM = 2,5; IC95%: 1,3-4,6).

**Conclusiones.:**

La salud mental de las madres puede poner en riesgo el bienestar socioemocional de sus hijos, lo que viene respaldado por la alta prevalencia y la asociación entre SDyA y DC encontrada. En México se necesita disponer de información fiable sobre el estado de salud mental de las mujeres y los niños de las tres ZP, para implantar medidas que amplíen su cobertura evaluativa y preventiva.

## Abstract (en)

**Objectives.:**

To estimate the prevalence of symptoms of depression and anxiety in mothers from three population areas in the state of San Luis Potosí, Mexico, and to analyze the association between these symptoms and the emotional health of their children.

**Methods.:**

Assessment was conducted on 173 mother–child pairs from three different population areas: urban, rural, and indigenous rural. Women's symptoms of depression and anxiety were evaluated, together with their children's behavioral problems.

**Results.:**

A high prevalence of symptoms of depression and anxiety was found in the three areas included. The proportion of depressive symptoms was highest in the urban and rural areas (38.7% and 38.6%, respectively). The highest prevalence of anxiety symptoms was observed in the rural area (31.8%). The strongest association between maternal anxiety and maternal depression was found in the rural area (odds ratio [OR] = 11.0; 95% CI: 1.3-95.5). The indigenous rural area showed the highest prevalence of behavioral problems among children (61%). An association between such problems and maternal anxiety (OR = 2.2; 95% CI: 1.1-4.3) and maternal depression (OR = 2.5; 95% CI: 1.3-4.6) was noted.

**Conclusions.:**

The mental health of mothers can put the social and emotional well-being of their children at risk. This conclusion is supported by the findings, namely high prevalences and an association between symptoms of anxiety and depression and behavioral problems. In Mexico, reliable information on the mental health status of women and children in the three population areas studied is needed in order to implement measures towards expanding coverage for evaluation and prevention.

Los trastornos del estado de ánimo y de ansiedad son dos de los padecimientos de salud mental con mayor prevalencia en adultos (1–2). Estos trastornos ocupan el primer y segundo lugar, respectivamente, en la distribución de frecuencias de las enfermedades con mayor número de años vividos con discapacidad (AVD) y de años de vida saludable perdidos (AVISA) a escala mundial. Su prevalencia suele ser más alta en mujeres que en hombres (3–4).

En México, los resultados de la Encuesta Nacional de Epidemiología Psiquiátrica (ENEP) señalan que los tres trastornos mentales más frecuentes padecidos alguna vez en la vida fueron los de ansiedad (14,3%) seguidos por los trastornos asociados con el abuso de sustancias (9,2%) y los del estado de ánimo (9,1%) (5). Al desagregar estos datos por sexo, los tres trastornos principales en las mujeres fueron las fobias (específicas y sociales) y el episodio depresivo mayor (5). Por otra parte, datos del Programa Nacional de Salud (2007–2012) indican que la depresión unipolar es una de las principales enfermedades responsables de AVISA en las mujeres. De acuerdo con lo anterior, las mujeres son más propensas a padecer estos trastornos y síntomas de depresión y ansiedad (6–7).

En otros estudios realizados con mujeres residentes de zonas marginadas de México el diagnóstico observado con mayor frecuencia fue el de los trastornos de ansiedad (16,4%) seguido por los afectivos (13,4%) (8). Según los años de vida ajustados por discapacidad (AVAD), la depresión mayor figura como la primera causa de discapacidad de las mujeres mexicanas (9).

Aunque la sintomatología de la depresión y de la ansiedad no reúna las características requeridas para su clasificación como trastorno, es importante su atención preventiva en los servicios de atención primaria, porque, si no se tratan, se está contribuyendo a que se agraven los síntomas, se complique el curso del tratamiento y repercutan en las relaciones interpersonales. Estos síntomas pueden ser factores de riesgo en el ambiente familiar de los padres y madres que los presentan, principalmente para las personas más cercanas como sus hijos y sus parejas. Por ejemplo, diversos estudios coinciden en señalar los efectos negativos de la depresión y la ansiedad parental en el desarrollo socioemocional del niño y las diversas implicaciones y costos inmediatos y futuros que tienen para la sociedad (10–16).

La mayoría de los estudios epidemiológicos realizados en México en que se analizan estos trastornos se han dirigido a evaluar a la población residente en zonas urbanas. Por ello, se carece de información suficiente y actual sobre la prevalencia de los trastornos mentales en mujeres y niños residentes en zonas rurales o indígenas. La ENEP, que se realizó hace quince años y que constituye la principal fuente de información de la salud mental en México, sólo aporta datos sobre adolescentes y adultos de zonas urbanas (17–18).

Ante esta situación, el presente estudio tuvo por objetivo estimar la prevalencia de síntomas asociados con trastornos de depresión y de ansiedad en mujeres residentes en zonas urbanas, rurales e indígenas del estado de San Luis Potosí, México, y averiguar si existe una asociación entre dichos síntomas y la presencia de dificultades socioemocionales de sus hijos.

## MATERIALES Y MÉTODOS

Se realizó un estudio observacional transversal. La selección de los participantes se realizó por muestreo probabilístico estratificado de tres zonas poblacionales del Estado de San Luis Potosí, México: Zona Urbana (ZU), Rural (ZR) y Rural indígena (ZRI). Inicialmente, se identificaron las escuelas primarias públicas ubicadas en cada una de las zonas de interés y de manera aleatoria se seleccionaron las escuelas participantes. Los tutores de los niños recibieron información sobre el objetivo del proyecto, la naturaleza voluntaria de la participación, así como sobre riesgos y beneficios del mismo. Además, se garantizó la confidencialidad de los datos personales y la devolución de los resultados de manera individual.

Los criterios de inclusión de las mujeres fueron: ser madres residentes en la zona del estudio, mayores de edad, no padecer trastornos mentales o estar tomando medicamentos psicoactivos, así como firmar una carta de consentimiento informado de su participación y la de sus hijos en el estudio. Los criterios de inclusión de los niños fueron: ser hijo de una de las mujeres participantes o estar bajo el cuidado de una de ellas, tener de 5 a 12 años de edad, ser estudiante de educación primaria, no padecer trastornos mentales clínicamente diagnosticados, y haber vivido desde el nacimiento en la misma zona poblacional. En el caso de las zonas indígenas, se incluyó el criterio de comprensión del idioma español tanto de las madres como de los niños.

En el estudio se utilizaron los instrumentos de evaluación que se describen a continuación.

### Depresión materna

Para evaluar la sintomatología depresiva de las mujeres se utilizó la escala de depresión de Kohout, et al. del Center for Epidemiologic Studies de 10 ítems (CES-D-10, por sus siglas en inglés) (19). El cuestionario consta de 10 ítems autoadministrados con los cuales se evalúa la frecuencia de los síntomas depresivos sentidos durante la semana anterior. El punto de corte para considerarlos positivos para depresión es mayor o igual a 10 puntos.

### Ansiedad materna

Para evaluar la ansiedad se empleó la escala de Ansiedad Manifiesta para Adultos *AMAS-A* (20), que consta de 30 ítems con los cuales se evalúan tres aspectos de la ansiedad en tres subescalas: 1) inquietud/hipersensibilidad, 2) ansiedad fisiológica, y 3) preocupaciones sociales/estrés. La puntuación total de la escala (ansiedad total) es la suma de las puntuaciones de las tres subescalas. Las puntuaciones finales se clasifican de menor a mayor gravedad: bajo, esperado, elevación ligera, clínicamente significativa, y extrema. Se consideraron puntuaciones elevadas las dos últimas, es decir, la clínicamente significativa y la extrema.

### Dificultades socioemocionales infantiles (DCE)

En este caso se utilizó la Escala de Capacidades y Dificultades (SDQ, por sus siglas en inglés) para evaluar las dificultades conductuales y emocionales de los niños (21). La SDQ es un cuestionario breve para detectar dificultades en el comportamiento, que está compuesto por 20 ítems divididos en cuatro subescalas: 1) síntomas emocionales, 2) problemas de conducta, 3) hiperactividad/ inatención, y 4) problemas con los compañeros. Cada subescala se obtiene sumando los cinco ítems respectivos, que determinan tres categorías: normal, límite o alto.

### Nivel socioeconómico (NSE)

Esta variable se midió con la regla AMAI 10 x 6 establecida por la Asociación Mexicana de Agencias de Investigación de Mercado y Opinión Pública (AMAI). Se trata de un índice que clasifica el nivel socioeconómico de los hogares en 6 niveles (A/B, C +, C, D +, D, y E). El nivel A corresponde al nivel socioeconómico más alto y el E, al más bajo. En esta escala se consideran el nivel de educación del jefe de la familia o de las personas que más contribuyen a los gastos del hogar, así como nueve características o posesiones del hogar (AMAI, 2008).

La administración y calificación de todos los instrumentos y cuestionarios fueron realizadas por psicólogos previamente capacitados y enmascarados (*blinded*) respecto a los trastornos de los participantes. Las aplicaciones se llevaron a cabo de forma individual en las aulas de las escuelas de los participantes y controlando los requisitos básicos de aislamiento, ausencia de ruido e iluminación apropiada.

Para llevar a cabo el análisis univariado, se calcularon medidas de tendencia central y de dispersión para las variables continuas y porcentajes para las variables categóricas. Para comparar porcentajes en el análisis bivariado se empleó la prueba de Chi cuadrada (χ2), y para comparar medias, el análisis de la varianza de un factor. Asimismo, se estimaron las razones de momios (RM u *odds ratios*) y sus intervalos de confianza del 95% (IC95%) como medida de la fuerza de la asocación entre las variables de interés. Todos los valores se calcularon a dos colas y el nivel de significación estadística alfa se fijó en 0,05. Los datos se analizaron con el paquete estadístico IBM SPSS Statistics versión 21.0 (22).

El estudio fue aprobado por la Comisión de Bioética S.L.P. de la Facultad de Medicina de la Universidad Autónoma de San Luis Potosí (UASLP) con el Expediente Ambiental-006/2010.

## RESULTADOS

En el estudio participaron en total 173 binomios madre-hijo de tres zonas poblacionales del estado de San Luis Potosí, México, distribuidos de la siguiente manera: 75 binomios en la ZU, 44 en la ZR y 54 en la ZRI (cuadro 1).

Las medias de la edad del total de las madres y de los niños participantes fueron 34,5 años (DE = 8,1) y 7,7 años (DE = 1,5), respectivamente. Los porcentajes de niños varones en la ZU, la ZR y la ZRI fueron, en orden decreciente, 62,7, 52,3 y 40,7%. El pocentaje de madres con NSE bajo fue mayor en la ZRI (96,3%), seguido por la ZR (29,5%) y la ZU (26,7%). Las puntuaciones medias por zona fueron las siguientes: ZU (132,1 ± 42), ZR (127,7 ± 52,6) y ZRI (53 ± 32,7). El análisis de la varianza realizado con las puntuaciones medias del NSE mostró que las diferencias detectadas entre la ZRI respecto a la ZU y la ZR fueron estadísticamente significativas (*F* = 60,61, gl = 2/170, *P* < 0,05).

Respecto a la depresión materna, la prevalencia de síntomas depresivos en la ZU fue 38,7%, mientras que en la ZR y en la ZRI fue 38,6 y 25,9%, respectivamente. Las puntuaciones medias por zona fueron las siguientes: ZU (8,2 ± 4,3), ZR (9,2 ± 6,6) y ZRI (7,0 ± 5,3). En el análisis de la varianza, las diferencias entre las medias de las tres zonas poblacionales no fueron estadísticamente significativas (*F* = 2,183, *gl* = 2/170, *P* = 0,116).

La prevalencia de ansiedad materna clínicamente relevante en el total de la muestra fue 22,5%. De manera independiente, en la ZR la prevalencia de casos (clínicamente relevantes y extremos) fue mayor (31,8%), seguida por la de la ZRI (20,4%) y la de la ZU (18,7%) (cuadro 2). Según el análisis por subescalas de la *AMAS-A*, en la ZRI se detectó el mayor porcentaje de casos por encima del punto de corte para la subescala de inquietud/hipersensibilidad (16,7%), mientras que el menor porcentaje de casos se encontró en la ZU (10,7%). Con las subescalas de ansiedad fisiológica y de preocupación social/estrés en la ZR se detectaron los porcentajes más elevados de casos (47,8 y 38,6%, respectivamente).

**CUADRO 1.** Características sociodemográficas de los participantes estratificados en tres zonas del estado de San Luis Potosí, México, 2013

Zona	Número de binomios madre-hijo	Edad madres $\bar{\text{x}}$ ± DE	NSE alto n (%)	NSE medio n (%)	NSE bajo n (%)	Edad niños $\bar{\text{x}}$ ± DE	Niños varones %
ZU	75	32,2 ± 5,7	2 (4,0)	52 (69,3)	20 (26,7)	7,0 ± 1,2	62,7
ZR	44	35,4 ± 7,0	5 (11,4)	26 (59,1)	13 (29,5)	8,4± 1,4	52,3
ZRI	54	37,4 ± 11,1	…	2 (3,7)	52 (96,3)	7,9 ± 1,5	40,7
Total	173	34,5 ± 8,1	7 (4,6)	80 (46,2)	85 (49,1)	7,7 ± 1,5	53,2

Zona urbana (ZU), Zona rural (ZR), y Zona rural indígena (ZRI). $\bar{\text{x}}$ ± DE, media ± desviación estándar; NSE, nivel socioeconómico.

**CUADRO 2.** Prevalencias de ansiedad materna en tres zonas del estado de San Luis Potosí, México, 2013

Zona	Ansiedad Total n (%)	Inquietud/ hipersensibilidad n (%)	Ansiedad fisiológica n (%)	Preocupación social/estrés n (%)
Urbana (n = 75)				
Baja	15 (20,0)	13 (17,3)	14 (18,7)	15 (20,0)
Esperada	18 (24,0)	21 (28,0)	23 (30,7)	14 (18,7)
Elevación leve	28 (37,3)	33 (44,0)	22 (29,3)	30 (40,0)
Clínicamente relevante	14 (18,7)	8 (10,7)	13 (17,3)	16 (20,0)
Extrema	…	…	3 (4,0)	…
$\bar{\text{x}}$ ± DE	17,0 ± 6,9	8,69 ± 3,3	4,2 ± 2,6	4,11 ± 1,8
Rural (n = 44)				
Baja	5 (11,4)	7 (15,9)	6 (13,6)	7 (15,9)
Esperada	7 (15,9)	12 (27,3)	9 (20,5)	1 (2,3)
Elevación leve	18 (40,9)	20 (45,5)	8 (18,2)	19 (43,2)
Clínicamente relevante	14 (31,8)	5 (11,4)	16 (36,4)	17 (38,6)
Extrema	…	…	5 (11,4)	…
$\bar{\text{x}}$ ± DE	18,9 ± 7,2	8,7 ± 3,6	5,6 ± 2,9	4,7 ±1,9
Rural indígena (n = 54)				
Baja	6 (11,1)	8 (14,8)	9 (16,7)	8 (14,8)
Esperada	20 (37,0)	20 (37,0)	18 (33,3)	8 (14,8)
Elevación leve	17 (31,5)	17 (31,5)	17 (31,5)	25 (46,3)
Clínicamente relevante	11 (20,4)	9 (16,7)	9 (16,7)	13 (24,1)
Extrema	…	…	1 (1,9)	…
$\bar{\text{x}}$ ± DE	17,1 ± 7,0	8,4 ± 3,4	4,4 ± 2,4	4,3 ± 1,8
Total (n = 173)				
Baja	26 (15,0)	28 (16,2)	29 (16,8)	30 (17,3)
Esperada	45 (26,0)	53 (30,6)	50 (28,9)	23 (13,3)
Elevación leve	63 (36,4)	70 (40,5)	47 (27,2)	74 (42,8)
Clínicamente relevante	39 (22,5)	22 (12,7)	38 (22,0)	46 (26,6)
Extrema	…	…	9 (5,2)	…
$\bar{\text{x}}$ ± DE	17,5 ± 7,0	8,6 ± 3,4	4,6 ± 1,8	4,30 ± 1,8

Se observó una asociación estadísticamente significativa entre las prevalencias de ansiedad y depresión de las madres participantes en las tres zonas (cuadro 3). En la ZR se estimó la mayor razón de momios (RM = 11; IC95%:1,3-95,5), seguida por la de la ZRI (RM = 9,0; IC95%: 1,8-45,7) y la de la ZU (RM = 5,4; IC95%:1,9-15,9). La RM en la población total fue 6,9 (IC95%: 3,1-15,3).

Según las puntuaciones obtenidas por los niños en la escala total de dificultades conductuales SDQ, 44,5% presentaron un alto riesgo de manifestar algún tipo de dificultad conductual. A este respecto, la mayor prevalencia de niños con puntuaciones altas en dicha escala se observó en la ZRI (61%), seguida por la ZU (44%) y la ZR (25%) (cuadro 4). Asimismo, en la ZRI las prevalencias de casos en las subescalas de síntomas emocionales, hiperactividad/ inatención y problemas con los compañeros fueron, respectivamente, 44,4; 35,2 y 64,8%. En las tres zonas, la proporción de niños con problemas con los compañeros fue alta, aunque las de la ZU y la ZR fueron las más elevadas (44 y 64,8%, respectivamente).

Por último, se encontró una asociación estadísticamente significativa entre las dificultades socioemocionales de los niños y la ansiedad materna en la muestra total (RM = 2,2; IC95%: 1,1-4,3; χ^2^_(172)_ = 5,10, *P* = 0,01) y con la depresión materna (RM = 2,5; IC95%: 1,3-4,6) (cuadro 5).

**CUADRO 3.** Asociación entre ansiedad y depresión materna estratificada en tres zonas del estado de San Luis Potosí, México, 2013

Zona	Depresión Materna	Ansiedad Materna			RM (IC95%)	Chi cuadrado	P
		(+)	(-)	Total			
Urbana (n = 75)	Positivo	23	6	29	5,4 (1,9-15,9)	10,3	< 0,001
	Negativo	19	27	46			
	Total	42	33	75			
Rural (n = 44)	Positivo	16	1	17	11,3 (1,3-95,5)	6,2	0,006
	Negativo	16	11	27			
	Total	32	12	44			
Rural indígena (n = 54)	Positivo	12	2	14	9,0 (1,8-45,7)	8,5	0,001
	Negativo	16	24	40			
	Total	28	26	54			
Total (173)	Positivo	51	9	60	6,9 (3,1-15,3)	25,7	< 0,001
	Negativo	51	62	113			
	Total	10	71	173			

(+), positivo; (-), negativo; RM, razón de momios; IC95%, Intervalo de confianza del 95%.

**CUADRO 4.** Prevalencia de dificultades conductuales en los niños estratificados en tres zonas del estado de San Luis Potosí, México

Zona	Escala Total Dificultades n (%)	Síntomas emocionales n (%)	Problemas de conducta n (%)	Hiperactividad/inatención n (%)	Problemas con compañeros n (%)
ZU (n = 75)					
Normal	26 (34,7)	33 (44,0)	28 (37,3)	42 (56,0)	21 (28,0)
Límite	16 (21,3)	19 (25,3)	14 (18,7)	18 (24,0)	11 (14,7)
Alta	33 (44,0)	23 (30,7)	33 (44,0)	15 (20,0)	43 (57,3)
ZR (n = 44)					
Normal	26 (59,1)	23 (52,3)	19 (43,2)	29 (65,9)	22 (50,0)
Límite	7 (15,9)	7 (15,9)	7 (15,9)	3 (6,8)	4 (9,1)
Alta	11 (25,0)	14 (31,8)	18 (40,9)	12 (27,3)	18 (40,9)
ZRI (n = 54)					
Normal	14 (25,9)	26 (48,1)	27 (50,0)	21 (38,9)	11(20,4)
Límite	7 (13,0)	4 (7,4)	8 (14,8)	14 (25,9)	8 (14,8)
Alta	33 (61,1)	24 (44,4)	19 (35,2)	19 (35,2)	35 (64,8)
Total (n = 173)					
Normal	66 (38,2)	82 (47,4)	74 (42,8)	92 (53,2)	54 (31,2)
Límite	30 (17,3)	30 (17,3)	29 (16,8)	35 (20,2)	23 (13,3)
Alta	77 (44,5)	61 (35,3)	70 (40,5)	46 (26,6)	96 (55,5)

Zona urbana (ZU), Zona rural (ZR), y Zona rural indígena (ZRI).

**CUADRO 5.** Asociación entre las dificultades socioemocionales infantiles y los síntomas de depresión y ansiedad materna del total de zonas estudiadas del estado de San Luis Potosí, México, 2013

Síntomas	Dificultades socioemocionales			RM (IC95%)
	Sí	No	Total	
Depresión Materna				
Positivo	44	16	50	2,2 (1,1-4,3)
Negativo	63	50	113	
Total	107	66	173	
Ansiedad Materna				
Positivo	72	30	102	2,5 (1,3-4,6)
Negativo	35	36	71	
Total	107	66	173	

RM, Razón de momios; IC95%, Intervalo de confianza del 95%.

## DISCUSIÓN

En este estudio, la prevalencia estimada de síntomas depresivos de las mujeres de la ZRI fue 25,9%, es decir, 1 de cada 4 presentaban síntomas, lo cual coincide con los resultados de un estudio llevado a cabo en México con una muestra de mujeres estudiantes universitarias mayores de 20 años de edad, en el cual se estimó una prevalencia de 27% de estos síntomas evaluados con el CESD-10 (23). En otro estudio realizado con mujeres de una zona rural de México la prevalencia fue 22,3% (24), en otros dos llevados a cabo con mujeres pobres de los Estados Unidos de América, 23,5% (25), y entre 26 y 33% (26), y en dos estudios más realizados en el mismo país con mujeres de bajo nivel socioeconómico dicha cifra fue 23,5% (25) y entre 26 y 33% (26). Sin embargo, en las mujeres de la ZU y ZR las prevalencias de estos síntomas fueron más altas (38,7 y 38,6%, respectivamente), lo que indica que en estas zonas casi 4 de cada 10 madres evaluadas padecen síntomas depresivos.

En cuanto a la presencia de síntomas de ansiedad materna, no se encontraron diferencias significativas en las puntuaciones medias entre las tres zonas. Sin embargo, cuando individualmente se contrastó cada zona con la media mexicana de referencia (12,4, DE = 6,6) (20), se encontró una diferencia significativamente por encima de este valor (*P* < 0,05) en las tres zonas. La prevalencia de síntomas de ansiedad estimada en la ZU fue 18,7%, similar a la encontrada en estudios previos realizados en zonas urbanas. De acuerdo con los datos de la ENEP (2003), la prevalencia de mujeres que presentaron alguna vez en su vida trastornos de ansiedad fue 18,5%. Los resultados de prevalencia de síntomas de ansiedad obtenidos en la ZR (31,8%), así como en la ZRI (20,4%), guardan similitud con los de otro estudio donde se evaluó una muestra urbana representativa de El Salvador en el cual la prevalencia de ansiedad e insomnio en mujeres fue 29,9% (27). Por el contrario, las estimaciones del presente estudio son mayores que las cifras notificadas en un estudio con mujeres residentes de zonas marginadas de México (16,4%) (8). Aunque este resultado es congruente con la prevalencia estimada en la ZU (18,4%), es la mitad que la de la ZR.

Los resultados obtenidos respecto a la asociación entre los síntomas de depresión y ansiedad coinciden con los publicados, ya que se observa una fuerte asociación entre dichos transtornos (28–29). Al analizar esta misma asociación entre zonas, se observó que en la ZR se estimó la más elevada. En esta zona, las madres que padecen síntomas depresivos son 11 veces más propensas a presentar síntomas de ansiedad, es decir, la posibilidad de presentar síntomas de ansiedad cuando se tienen síntomas depresivos es de 11 veces mayor en la ZR. Del mismo modo, en la ZRI es 9 veces más alta, en la ZU, 5,4 veces, y en la muestra total, casi 7 veces más elevada. Estos datos también sugieren que la zona de residencia representa un factor de riesgo para las personas que padecen síntomas depresivos, ya que principalmente las zonas rurales son las de mayor riesgo por ser más pobres, por carecer de apoyo social, porque en ellas los servicios de salud son escasos o están alejados y por la falta de asistencia en salud mental y desconocimiento.

Llama la atención que en las madres de la ZRI se estimaron las prevalencias de síntomas de depresión y ansiedad más bajas. Sin embargo, se ha observado que la satisfacción percibida y los fundamentos del bienestar en las mujeres pueden variar entre zonas poblacionales en México (30). En cuanto a este punto, en otro estudio se señala que ya se había propuesto con anterioridad que en la población rural mexicana, donde el sufrimiento se considera parte del papel de género femenino, los síntomas depresivos podrían no ser un buen indicador de depresión clínicamente relevante (24). En ello estriba la necesidad de profundizar en aspectos de género a fin de comprender y responder a las necesidades de las mujeres originarias de México.

Por otra parte, los resultados de esta investigación indican que en la muestra total el riesgo de presentar dificultades conductuales de los niños cuyas madres padecen síntomas depresivos fue dos veces más alto que el de los niños cuyas madres no los padecen, y 2,5 veces más cuando sus madres sufren síntomas de ansiedad. En algunos estudios se consigna que los trastornos de depresión y ansiedad parental pueden interferir en la dinámica familiar, en la relación matrimonial (31), en la crianza (32), así como en el desarrollo cognitivo y emocional de los niños (13–16). En este estudio también se afirma que 6 de cada 10 niños de la ZRI presentan dificultades conductuales, principalmente síntomas emocionales, hiperactividad/inatención y problemas con los compañeros. Aunque en las tres zonas la prevalencia de niños con problemas con los compañeros fueron altas, las más elevadas se estimaron en la ZU y la ZR (44 y 64,8%, respectivamente). Este tipo de conductas se encuentran fuertemente asociadas con síntomas relacionados con trastornos de conducta, depresión, ansiedad, déficit de atención, comportamientos de acoso escolar, y hostigamiento como el *bullying*, cuya frecuencia está aumentando en estas poblaciones a nivel mundial. Llama la atención que 44,5% de la muestra total presentó un alto riesgo de manifestar algún tipo de dificultad socioemocional, lo cual señala un foco al que debe prestarse especial atención, porque traduce la existencia de factores que están poniendo en riesgo la salud mental y social de estos niños.

Se ha subrayado que los trastornos de la salud mental en los niños representan una carga importante de enfermedad y generan un impacto en su funcionamiento cotidiano y calidad de vida y en el de sus familias. En este sentido, la Organización Mundial de la Salud (OMS), en el Objetivo 3 de su Plan de Acción Sobre Salud Mental 2013–2020, insiste en aplicar estrategias de promoción y prevención en materia de salud mental, y señala que es a los gobiernos a quienes corresponde utilizar la información y los datos generados sobre los factores de riesgo y de protección, para emprender acciones destinadas a prevenir los trastornos mentales y a proteger y promover la salud mental en todas las etapas de la vida, al tiempo que subraya que hasta 50% de los trastornos mentales que afectan a los adultos empiezan antes de los 14 años de edad (33).

Por otra parte, la OMS destaca que una contribución esencial para el logro de los Objetivos de Desarrollo del Milenio (ODM) es mejorar la salud de los niños y sus madres y ocuparse de las cuestiones que afectan a su salud (34). En México, aunque ha habido un fuerte avance en algunos de estos objetivos, aún se han de realizar esfuerzos mantenidos en el tiempo y homogéneos para fortalecer el desarrollo y empoderamiento de las mujeres, porque a menudo son las responsables de la crianza de sus hijos y cada vez más lo hacen solas. Este hecho es una de las muchas razones que subrayan la importancia de la situación socioeconómica de las mujeres en el bienestar de las generaciones futuras (35). No obstante, las nuevas investigaciones que se lleven a cabo han de incorporar aproximaciones psicosociales en la prevención de la depresión en mujeres de alto riesgo y prestar especial atención a las necesidades de tratamiento de las mujeres deprimidas y de sus familias. Los niños y adolescentes con trastornos mentales deben ser objeto de intervenciones tempranas científicamente contrastadas de carácter no farmacológico, ya sean psicosociales o de otra índole, realizdas en el ámbito comunitario y en ellas han de evitarse la institucionalización y la medicalización (33).

Cabe concluir que la salud mental de las madres de las tres zonas estudiadas y el bienestar socioemocional de sus hijos se encuentran comprometidos. Las ZU y ZR presentan la mayor prevalencia de madres con SDyA. En los niños con madres que tienen síntomas depresivos el riesgo de manifestar DCE es dos veces más alto que el de aquellos cuyas madres no los padecen y 2,5 veces más elevado en los hijos de madres con síntomas de ansiedad. Fue en la ZRI donde prevalencia de niños con dificultades socioemocionales fue mayor. A pesar de que existen pocas estrategias y programas enfocados a prevenir y atender dichas condiciones, hay barreras que obstaculizan esta atención. Una de las principales es la falta de datos e información relacionados con los trastornos mentales en poblaciones rurales e indígenas, lo cual convierte en indispensable la necesidad de instrumentalizar medidas que amplíen la cobertura evaluativa y preventiva, y privilegien modelos comunitarios, sobre todo en los grupos sociales más vulnerables y marginados.

## Agradecimiento.

Los autores agradecen a la UASLP y al CONACYT su apoyo y financiamiento.

## Financiamiento.

Este estudio fue financiado por el fondo del CONACYTCiencia Básica No. 133149.

## Conflictos de interés.

Ninguno declarado por los autores.

## Declaración.

Las opiniones expresadas por los autores son de su exclusiva responsabilidad y no reflejan necesariamente los criterios ni la política de la Organización Panamericana de la Salud o de la *RPSP/PAJPH.*
